# The influence of the marker set on inverse kinematics results to inform markerless motion capture annotations

**DOI:** 10.1038/s41598-025-97219-5

**Published:** 2025-04-25

**Authors:** Marion Mundt, David Pagnon, Steffi Colyer

**Affiliations:** 1 Nutrition and Health Innovation Research Institute, Edith Cowan University, Joondalup, WA Australia; 2 UWA Tech & Policy Lab, The University of Western Australia, Crawley, WA Australia; 3 Centre for the Analysis of Motion, Entertainment Research and Applications, University of Bath, Bath, UK; 4 Department for Health, University of Bath, Bath, UK

**Keywords:** Motion capture, Musculoskeletal modelling, Biomechanics, Computer vision, Pose estimation, Musculoskeletal system, Biomedical engineering

## Abstract

Markerless motion capture has the potential to enable biomechanical analyses without specialised, high-cost equipment. However, the comparability of many markerless motion capture frameworks with the most used marker-based method is limited. One reason for this is the lack of high-quality, biomechanically-informed datasets that are needed to train markerless models. This study aimed to inform the development of such a dataset by systematically analysing the agreement between a gold-standard marker set and a reduced number of markers to solve inverse kinematics (IK). We analysed the impact of different marker positions on the IK solution using an OpenSim lower body model with real and synthetic data of running, walking and counter movement jumps. We found that one mid-segment marker in addition to two anatomical markers per segment result in the best agreement to a gold-standard marker set. The results for real and synthetic data across all movements were similar, with synthetic data showing slightly better agreement with a reduced number of markers (root mean squared error 1.55–8.27^°^ real data, 1.27–7.79^°^ synthetic data), likely due to limited soft tissue artefacts and missing human error in marker placement. These findings can support the development of a dataset to retrain markerless models incorporating biomechanical knowledge.

## Introduction

Computer-vision-based pose estimation models for the automation of markerless motion capture show a great potential to change the field of biomechanics. Using simple 2D video cameras outside the laboratory without the need for any markers placed on a patient’s or athlete’s body will make motion capture more accessible in everyday clinical and sports environments and enable medical practitioners and coaches to quickly access information on joint angles and other relevant parameters during a testing or training session. However, current pose estimation models have not been developed specifically for biomechanics, therefore not providing the necessary accuracy to perform motion analysis at a clinical or high-performance sport standard^1–4^.

Large databases of images are used to train pose estimation models to automatically detect joint centres and anatomical landmarks, so called keypoints. These models and datasets have multiple limitations for biomechanics applications, including: (1) images have been annotated by crowd-workers without biomechanics knowledge, limiting the accuracy of annotations; (2) the number of keypoints in each image is typically limited to two keypoints to describe a segment, allowing for only pseudo-3D reconstruction of a movement; (3) a pose is detected frame-by-frame, resulting in noisy keypoint estimates due to the lack of temporal information^1,3,5^.

Marker-based motion capture, the most commonly used method in biomechanics, also presents noise and erroneous anatomical landmarks due to soft tissue artefacts and misplaced anatomical markers^6,7^. To overcome these limitations, unconstrained inverse kinematics (also referred to as direct kinematics) have been replaced by constrained inverse kinematics (IK). While the unconstrained approach simply uses anatomical markers for the direct calculation of joint angles, the constrained approach solves a global optimisation problem to minimise the weighted least-squares distance between experimental markers and virtual markers placed on a computational rigid body model^8^. While unconstrained models can result in unphysiological behaviour like changing segment lengths, disconnected bodies, and unrealistic joint angles, constrained models prohibit this behaviour. On the downside, the results depend on the definition of the model^8^, marker registration and model scaling^9,10^, and inverse kinematics weights^7,11^.

On top of the described settings of the computational model used for IK calculations, the marker set used during data capture has an impact on the results^11–13^. Theoretically, three markers per segment are needed to fully define a rigid segment’s degrees of freedom. If this segment is connected to another segment by a ball or hinge joint, the number of markers is reduced to two or one. However, it has been shown that using redundant markers can improve IK results by minimising the influence of soft tissue artefacts^14^, which depend on marker location^15^. Marker clusters placed mid-segment have proven to be the most effective solution^16^. The choice of marker set is particularly important for data collection in sports, where large soft tissue movements can be expected dependent on the movement performed^14,17^.

Recent studies have used IK with models with constrained degrees of freedom (e.g. a knee joint modelled as a hinge joint) to support markerless motion capture^18–21^. The results showed improved kinematic results, which is in line with studies that reported highly reproducible kinematics with constrained models and sparse marker sets^22^. To accurately model pathological movement or movements with large ranges of motion in the minor motion axes, models with less constraints are needed. However, with sparse keypoints, as is current practice in markerless motion capture, joint angles cannot be described in all degrees of freedom using unconstrained models, and are more likely to be affected by measurement errors when using an IK approach. In an attempt to overcome this limitation, artificial neural networks have been trained to estimate a rich marker set from sparse keypoint estimations. However, this approach is largely dependent on the underlying dataset used for training the artificial neural network models and only performs well on movements that are part of the training dataset^4,23^.

We aim to take a different approach and develop an image dataset containing biomechanically-informed annotated images with sufficient keypoints per segment to define 3D hip, 3D knee, and 2D ankle joint angles compatible with marker-based motion analysis pipelines. As a first step towards this aim we investigate in this study how many markers/keypoints are necessary to achieve similar IK results compared with a gold-standard marker set and where these markers should be placed. To achieve this, we first answer these questions using a motion capture dataset and, in a next step, verify if these results are valid for a synthetic dataset. We hypothesise that there will be differences between the real and synthetic data due to the lack of soft tissue movements in animation data.

## Results

We investigated the influence of six marker combinations (Table 1) on the resulting IK joint angles of the lower limbs for measured data (bioCV) and synthetic data (BEDLAM) for running (RUN), walking (WALK), and counter movement jumps (CMJ).

**Table 1:** Markers used for inverse kinematics (number total).

	M01	M02	M03	M04	M05	M06
Ground-pelvis	Constraining pelvis movement relative to the ground to 30^*°*^ in each axis					
Pelvis	ASIS, PSIS, ILCREST (6)	ASIS, PSIS (4)	ASIS, PSIS (4)	PSIS, MIDHIP (3)	PSIS, MIDHIP (3)	MIDHIP (1)
Hip joint	Custom joint with three unconstrained degrees of freedom					
Thigh	KNEE_MED, KNEE_LAT 4 mid-segment (6)	KNEE_MED, KNEE_LAT 1 mid-segment (3)	KNEE_MED, KNEE_LAT (2)	HJC, KJC, 2 mid-segment (4)	HJC, KJC, 1 mid-segment (3)	HJC, KJC (2)
Knee joint	Custom joint with three degrees of freedom constrained to 120^°^ flexion, 10^°^ extension, 45^°^ internal rotation, 30^°^ external rotation, 15^°^ abduction, 15^°^ adduction, 20 mm anterior-posterior translation					
Shank	MAL_MED, MAL_LAT, 4 mid-segment (6)	MAL_MED, MAL_LAT, 4 mid-segment (3)	MAL_MED, MAL_LAT, (2)	AJC, 2 mid-segment (3)	AJC, 1 mid-segment (2)	AJC (1)
Ankle joint	Custom joint with one unconstrained degree of freedom					
Subtalar joint	Custom joint with one unconstrained degree of freedom					
Foot	HEEL, MTP1, MTP5, TOE (4)	HEEL, MTP1, MTP5, TOE (4)	HEEL, MTP1, MTP5, TOE (4)	HEEL, MTP1, MTP5 (3)	HEEL, MTP1, MTP5 (3)	HEEL, MTP1, MTP5 (3)

### BioCV dataset

The root mean squared error (RMSE) and agreement between IK results varied depending on the marker set (Supplementary Table 1). Reducing the number of mid-segment markers per segment from 4 (M01) to 1 (M02) resulted in a mean RMSE of 1.55 ± 0.80^°^ and coefficient of multiple correlation (CMC) of 0.926 indicating good agreement. A marker set without any mid-segment markers (M03) resulted in an RMSE of 3.11 ± 1.48^°^ and CMC of 0.786. The results for M04 (joint centres, two mid-segment markers; RMSE 2.06 ± 0.94°, CMC 0.931) and M05 (joint centres, one mid-segment marker; RMSE 2.44 ± 1.05°, CMC 0.916) were similar to M03 (medial/lateral markers, no mid-segment markers). Without any mid-segment marker and only joint centres (M06), the RMSE increased to 8.27 ± 3.33^°^ and the CMC value decreased to 0.783, indicating moderate agreement.

**Fig. 1:**
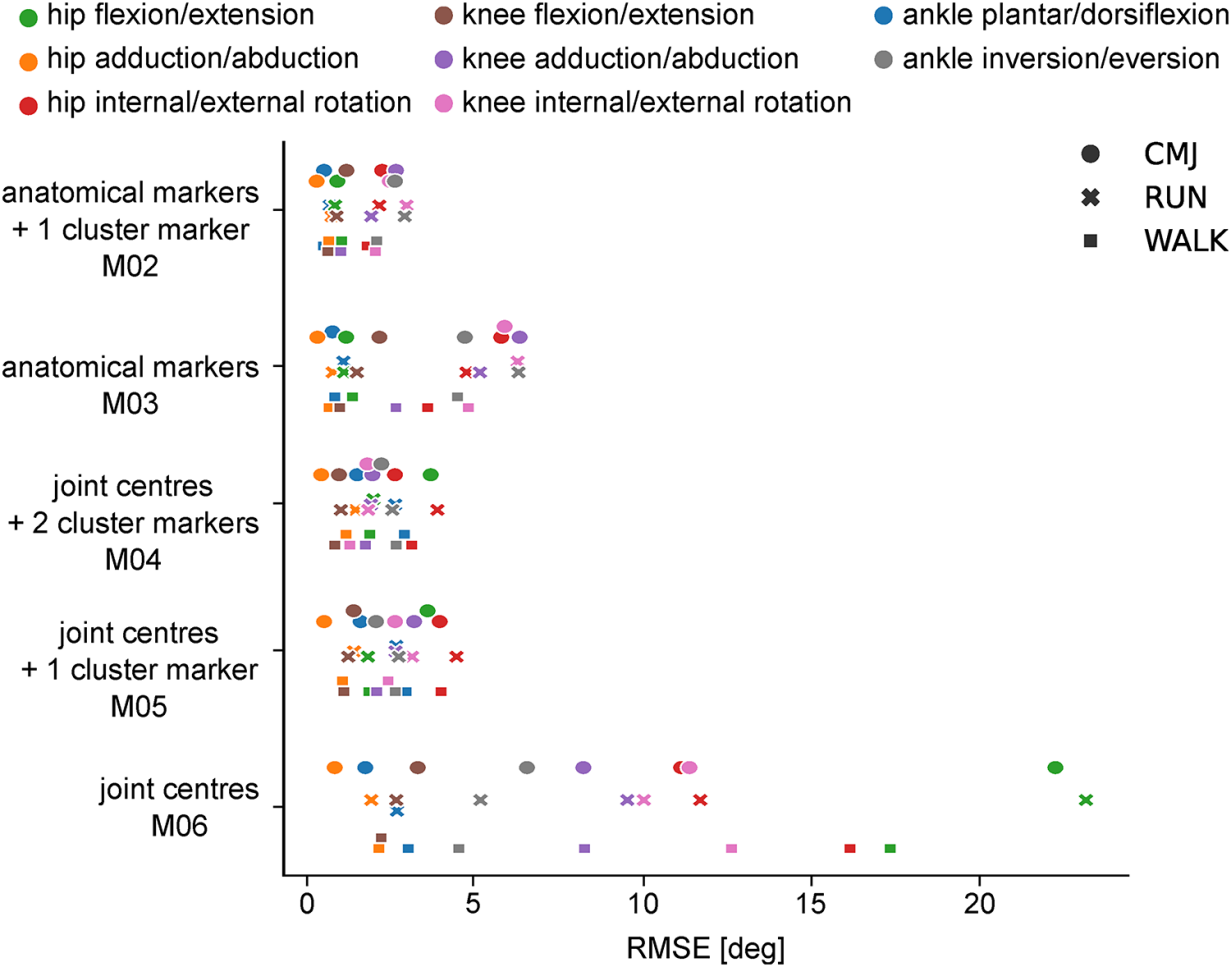
Mean RMSE of all marker sets per movement and angle in the bioCV dataset.

The RMSE for all joint angles across all movements was smaller than 5^°^ and CMC values indicate moderate to good agreement, if each segment was described by at least three markers including a mid-segment marker (M02, M04, M05) (Fig. 1, Supplementary Table 1) across all joint angles besides knee abduction/adduction that showed fair agreement for counter movement jumps. Although all segments were linked in the OpenSim model and the knee joint motion was constrained, the reduction of markers for the thigh and shank segments in M03 (medial/lateral markers, no mid-segment markers) resulted in an increased RMSE and poor to moderate CMC values for the knee adduction/abduction, hip and knee internal/external rotation and ankle inversion/eversion across all movements. The sparse marker set used in M06 (joint centres, no mid-segment markers) resulted in large RMSE values ranging between 2 and 23°. The hip joint angle was particularly affected because the pelvis movement in the linked system was described by a single marker and loose segment constraints only, which allowed for increased pelvis rotation in all three movement planes. The CMC values indicated overall poor correlations between joint angle waveforms when using this marker set besides hip adduction/abduction, knee flexion/extension and ankle plantar/dorsiflexion.

These findings were supported by the linear mixed effect models and effect sizes for ROM and RMSE (Supplementary Table 2, Supplementary Table 3). A significant influence of marker set but not movement (*p* < 0.001) was found for all joint angles ROM and RMSE. For the RMSE, large to very large effect sizes were found between all marker combinations and M06 (joint centres, no mid-segment markers). The difference in RMSE between M02, M04, and M05 (at least one mid-segment marker) was minor, showing trivial to small effect sizes for all joint angles but ankle plantar/dorsiflexion. The effect sizes between M02, M04, and M05 in comparison with M03 (medial/lateral markers, no mid-segment marker) showed larger effects for all joints but the hip. For the ROM, only M06 (joint centres, no mid-segment marker) showed large to very large effect sizes in comparison with all other models. The ROM was overall less affected by different marker sets than the RMSE.

**Fig. 2:**
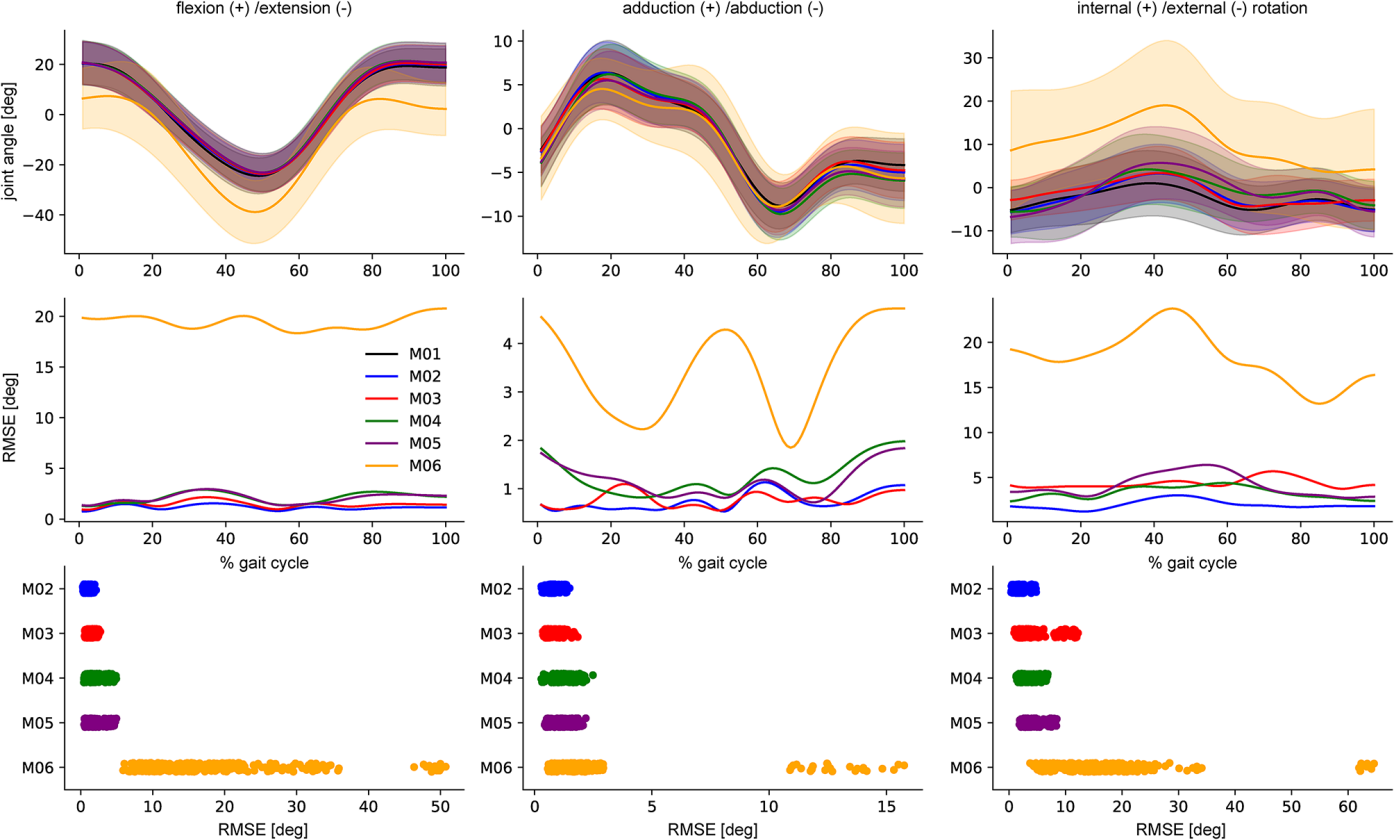
Mean and standard deviation of the 3D hip joint angle IK time series for the six marker sets investigated (top), the RMSE for each time step using the full marker set (M01) as reference (middle), and the mean RMSE for each marker set (bottom) exemplary displayed for walking using the bioCV data.

The resulting IK 3D hip angle time-series are displayed in Fig. 2 for walking as an example. The differences in hip flexion/extension angle between all marker sets but M06, where the joint angle showed a distinct offset, are small (M02-M05 RMSE < 1.91, CMC > 0.996, M06 RMSE = 17.26, CMC = 0.723). A similar but less pronounced pattern was found for hip adduction/abduction; for all marker sets but M06 the joint angles were similar (RMSE < 1.13, CMC > 0.987, M06 RMSE = 2.20, CMC = 0.905). Internal/external rotation was most affected by different marker sets. Missing mid-segment markers resulted in larger differences in joint angles waveforms (M03 RMSE = 3.64, CMC = 0.676; M06 RMSE = 16.07, CMC = NULL) compared with marker sets with mid-segment markers (M02 RMSE = 1.85, CMC = 0.924; M04 RMSE = 3.17, CMC = 0.827; M05 RMSE = 4.04, CMC = 0.802) (Supplementary Table 1).

### BEDLAM dataset

The synthetic data showed a similar pattern to the bioCV data (Supplementary Table 4): the lowest error was found in M02 (medial/lateral markers, one mid-segment marker; RMSE = 1.37 ± 0.63^°^, CMC = 0.961) and the highest in M06 (joint centres, no mid-segment marker; RMSE = 7.79 ± 3.50^°^, CMC = 0.657) with the other three marker sets performing similarly (M03 RMSE = 2.34 ± 1.17^°^, CMC = 0.865; M04 RMSE = 1.99 ± 1.17^°^, CMC = 0.889; M05 RMSE = 2.48 ± 1.38^°^, CMC = 0.847).

**Fig. 3:**
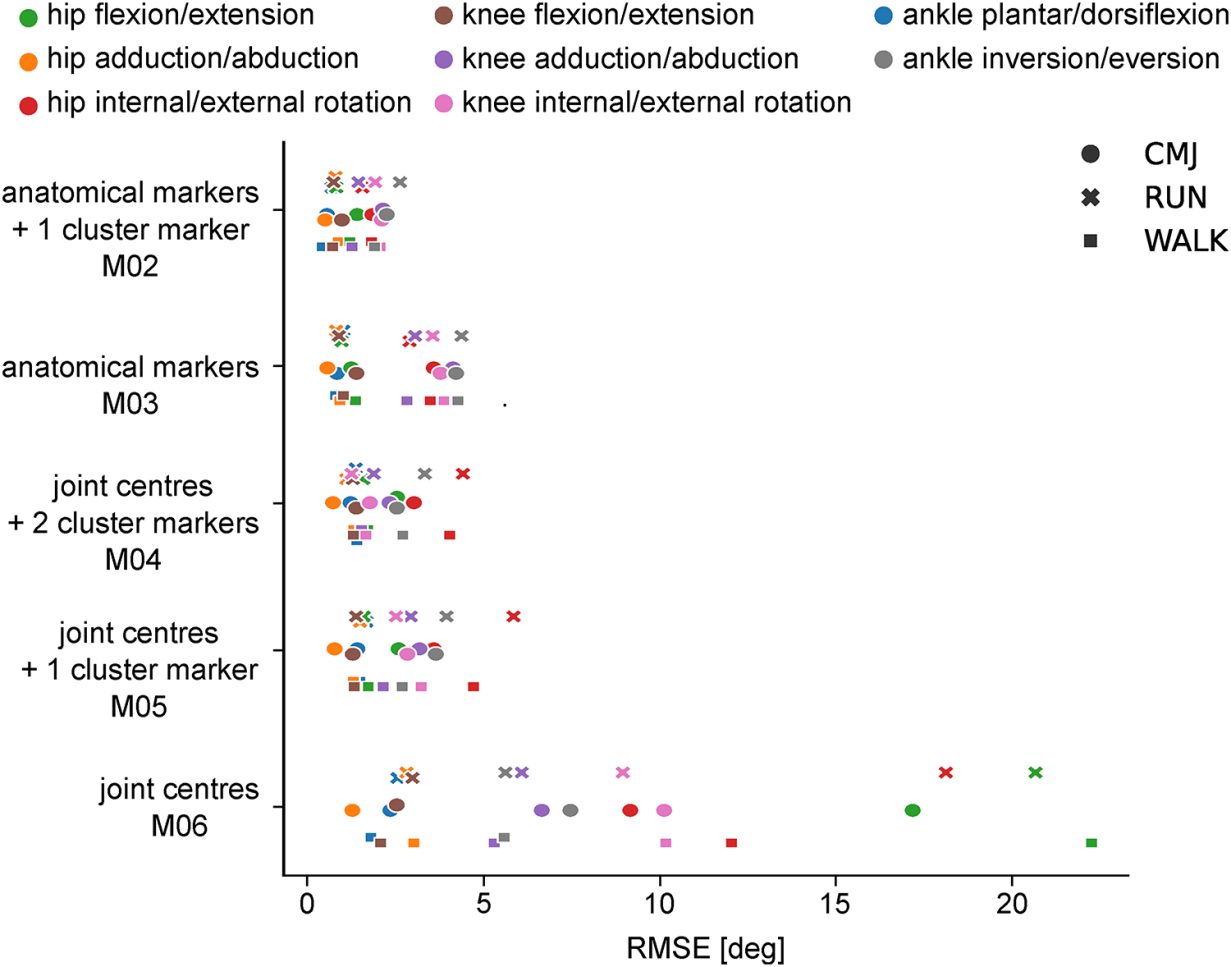
Mean RMSE of all marker sets per movement and angle in the synthetic BEDLAM dataset.

For all marker sets but M06 (joint centres, no mid-segment marker) the RMSE for joint angles was smaller than 5^°^ besides hip internal/external rotation for running in M05 (Fig. 3, Supplementary Table 4). However, CMC values indicated good agreement throughout all joint angles for M02 only (Supplementary Table 4). Hip internal/external rotation, knee adduction/abduction, knee internal/external rotation, and ankle inversion/eversion showed moderate to poor agreement for M03, M04, and M05 (Supplementary Table 4). For M06 (joint centres, no mid-segment marker), large RMSE values of up to 22° can be found with the hip joint angles showing the poorest result. The CMC values indicate overall poor agreement between joint angle waveforms when using this marker set besides hip adduction/abduction, knee flexion/extension and ankle plantar/dorsiflexion; similarly to the bioCV dataset (Supplementary Table 4).

The linear mixed effect model and effect sizes for ROM and RMSE showed a similar trend to the bioCV dataset. A significant influence of marker set and movement (*p* < 0.001) was found for all joint angles ROM and RMSE (Supplementary Tables 5, 6). For the RMSE, large to very large effect sizes were found between all marker sets and M06. The difference in RMSE between M02, M04, and M05 (at least one mid-segment maker) was minor, showing trivial to small effect sizes for all joint angles but ankle plantar/dorsiflexion. The difference between M02 and M04 in comparison with M03 (medial/lateral markers, no mid-segment marker) was smaller in this dataset than in the bioCV data, showing moderate effect sizes for knee adduction/abduction, internal/external rotation, and both ankle joint angles. For M05 (joint centres, one mid-segment marker), only ankle plantar/dorsiflexion showed moderate effect sizes when compared with M03. The type of movement did not show a consistent effect on the RMSE (Supplementary Table 5). The ROM is only affected when using M06, showing large to very large effect sizes in comparison with all other models in hip and knee internal/external rotation and ankle inversion/eversion only for CMJ (Supplementary Table 6).

**Fig. 4:**
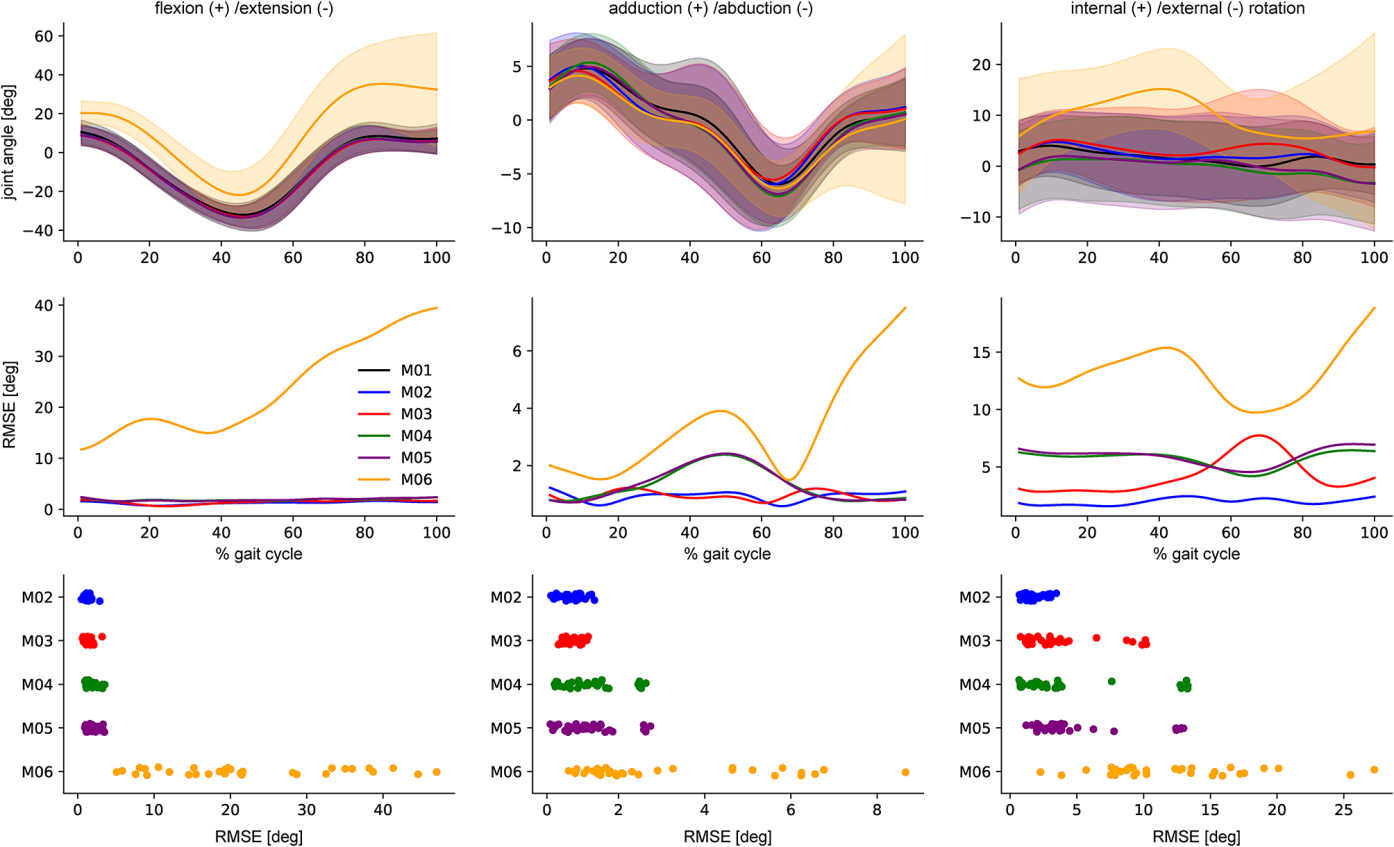
Mean and standard deviation of the 3D hip joint angle IK time series for the six marker sets investigated (top), the RMSE for each time step using the full marker set (M01) as reference (middle), and the mean RMSE for each marker set (bottom) exemplary displayed for walking using the synthetic BEDLAM data.

The resulting IK 3D hip angle time-series for walking are displayed in (Fig. 4). The differences between all six marker sets on the hip flexion/extension angle were small (RMSE < 1.74, CMC > 0.996), besides for marker set M06 (joint centres, no mid-segment marker) (RMSE = 22.27, CMC = 0.716); the joint angle showed a distinct offset. All marker combinations but M06 showed similar results for hip adduction/abduction (RMSE < 1.32, CMC > 0.970; M06 RMSE = 3.03, CMC = 0.776). Internal/external rotation was strongly affected by different marker sets. Only M02 (medial/lateral markers, one mid-segment marker) showed reasonable comparability to the ground-truth (RMSE = 1.83, CMC = 0.931), while the waveform of M03 (medial/lateral markers, no mid-segment marker) was distinctly different (RMSE = 3.50, CMC = 0.762). Using joint centres and mid-segment markers as inputs resulted in similar waveforms that are different to the ground-truth (M04 RMSE = 4.05, CMC = 0.325; M05 RMSE = 4.73, CMC = 0.365). M06 resulted in a different waveform with a distinct offset (RMSE = 12.05, 200 CMC = 0.196) (Supplementary Table 4).

## Discussion

This study aimed to inform the development of a synthetic training dataset for biomechanically informed pose estimation. For this purpose, we assessed the minimum number of markers and marker locations that are necessary to accurately determine lower body joint angles using an IK approach. We used the same scaled models throughout the analysis to ensure that we exclude the influence of the anatomical model and joint constraints that have shown to have a large effect on joint kinematics calculations^8,13^ and the model scaling, which is another source of uncertainty^10^. With this approach, we only include errors due to the marker set.

The RMSE for the BEDLAM dataset was slightly lower than for the bioCV dataset and the effect sizes for RMSE and ROM were also smaller than for bioCV. However, the results followed the same pattern. The smaller errors can likely be attributed to limited soft tissue movements^24^, no camera reconstruction errors^25^, and no human error in marker placement in the synthetic data, that can cause marker errors of 5–10 mm^7,17^ and 1–5 mm^25^, respectively.

Similar to previous studies^12^, we found good agreement by means of large CMC values for flexion/extension movements, and poorer reliability for the other motion planes. The determination of adduction/abduction and internal/external rotation movements is difficult using external markers due to soft tissue artefacts and small ranges of motion. In these minor motion planes the noise in the signal is large, leading to poor results^7,17^. Therefore, these angles are regularly excluded from analyses and the knee joint is modelled as a hinge joint^22^.

The typical uncertainty in joint angle calculation because of soft tissue artefacts has been defined to be 5^°^^7,22^. This threshold was exceeded when using marker sets with less than three markers per segment only (M03 and M06) and limited joint constraints. While model constraints can restrict the movement to reasonable ranges, these constraints have to be chosen carefully, as they have a large impact on the result. A constraint that is selected too tight will not allow for modelling the real movement, while a constraint that is selected too loose with insufficient markers controlling the IK solution will result in unphysiological movements. Due to the loose constraints of the knee joint, mainly the adduction/abduction and internal/external rotation were affected by a reduced number of markers. The hip joint angles in M06 (joint centres, no mid-segment marker) showed a larger effect because of missing pelvis markers. We did not find large differences between movements for any marker combination. This could either be the case because the fast movements, RUN and CMJ, were not fast enough to create meaningful differences compared with WALK, or soft tissue artefacts do not have a large impact on the markers used in this study.

Our findings are supported by another study that found mid-segment markers to not impact the analysis of walking as long as medial and lateral markers are present^12^, with similar reported error (*<* 5^°^). They also reported large errors (*<* 16^°^) for the plug-in-gait marker set, that contains a single marker per joint and no mid-segment markers, which is in line with the error we found using only joint centres as input (M06). We extended the previous study to faster movements different from walking and used common pose estimation keypoints instead of marker sets commonly used in biomechanics.

The main limitation of this study is the missing true ground-truth data. We have chosen a gold-standard biomechanics lower-body marker set as reference, however, this is also impacted by scaling and marker weights. To be able to use markers that are similar to pose estimation keypoints (M06), we had to constrain the pelvis movement. This constraint (± 30^*°*^) was modelled between the ground and pelvis and, therefore, out-of-plane movements could not be modelled adequately. The constraint was chosen rather loose, to allow for capturing realistic motion for all movements analysed but at the same time resulted in increased hip joint angles for movements with small pelvis motion for M06.

In summary, a marker set containing only joint centres, as is the current standard in pose estimation models, was not sufficient to drive IK simulations with an acceptable degree of accuracy. A rigid body’s position and orientation in 3D space can be fully described by three non-collinear points. Although an IK solution is a global optimisation that is controlled by markers, joints, and model constraints, the best solution was still achieved using three markers per segment. Fewer markers could be used with a more constrained model. However, redundant markers allow for a better definition of rotation axes and greater certainty in the IK solution. The location of the markers, especially in synthetic data that contains less soft tissue artefacts, was less important.

This information can be seen as a first step towards developing a synthetic dataset for retraining a biomechanically-informed pose estimation model. Multiple additional steps are necessary to achieve accurate markerless motion capture: First, the accuracy of existing synthetic datasets needs to be analysed with regard to the validity of skeletal and skin movement. Second, the detection accuracy of the markers identified in this study in real videos needs to be evaluated to finally evaluate the accuracy of biomechanical parameters such as joint angles determined by a retrained pose estimation model.

## Methods

This study was undertaken on two datasets: a motion capture dataset, bioCV^26^, and a synthetic dataset, BEDLAM^24^.

### BioCV dataset

The publicly available bioCV dataset^27^ contains ten running (RUN), walking (WALK), and counter movement jump (CMJ) trials performed by 15 healthy volunteers (7 males, 8 females, 26 ± 5 years, 73.7 ± 14.7 kg, 1.73 ± 0.11 m). Motion capture data were collected using a 15-camera optical motion capture system (Qualisys Oqus, 280 200 Hz) that had been calibrated as per the manufacturer’s guidelines. The study was conducted according to the guidelines of the Declaration of Helsinki, and approved by the Institutional Review Board (or Ethics Committee) of the University of Bath (EP1819052 25/07/19). Participants provided written informed consent prior to taking part in the study.

**Fig. 5:**
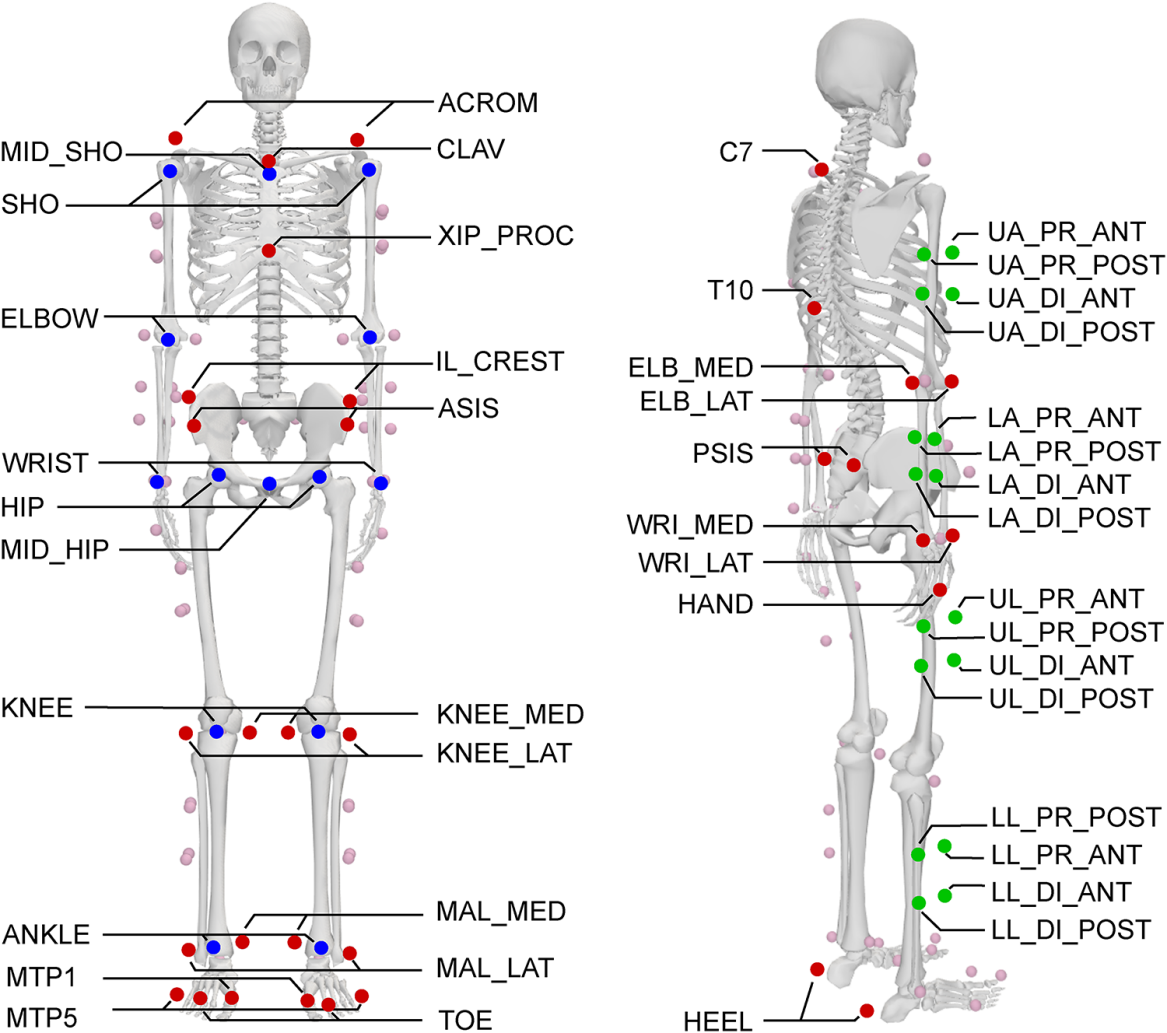
Overview of the marker set, and joint centres used to calculate the inverse kinematics. Anatomical markers are displayed in red, mid-segment markers in green and joint centres and mid-points in blue. Note that only labelled markers are displayed in colour. Modified image generated in OpenSim v4.5 (https://simtk.org/;^37^).

Each participant was equipped with a full-body marker set comprising 38 individual markers (anatomical landmarks) and eight 4-marker clusters (mid-segment) (Fig. 5). Since we only analysed movements that are predominantly defined by the lower body, we only considered lower body markers. Joint centres of knee and ankle were calculated as the mid-point between their respective medial and lateral marker, and the hip joint centres were calculated using regression equations^28^. The mid-point between the hip joint centres was also calculated as this is a common pose estimation keypoint (MID_HIP in Fig. 5). All CMJ trials were cropped from first movement to stable, running and walking trials were cropped to single strides (touchdown to subsequent touchdown with the same foot). Events in the bioCV dataset had been manually labelled previously^1^.

### BEDLAM dataset

The BEDLAM dataset contains animated synthetic but realistic scenes of people performing a multitude of different movements^24^. People are described by an SMPL-X mesh, which can be adjusted to take almost any human shape and posture. These meshes are animated and then rendered with various skin textures and clothing. Finally, they are put in a virtual environment, along with other synthetic people.

**Fig. 6:**
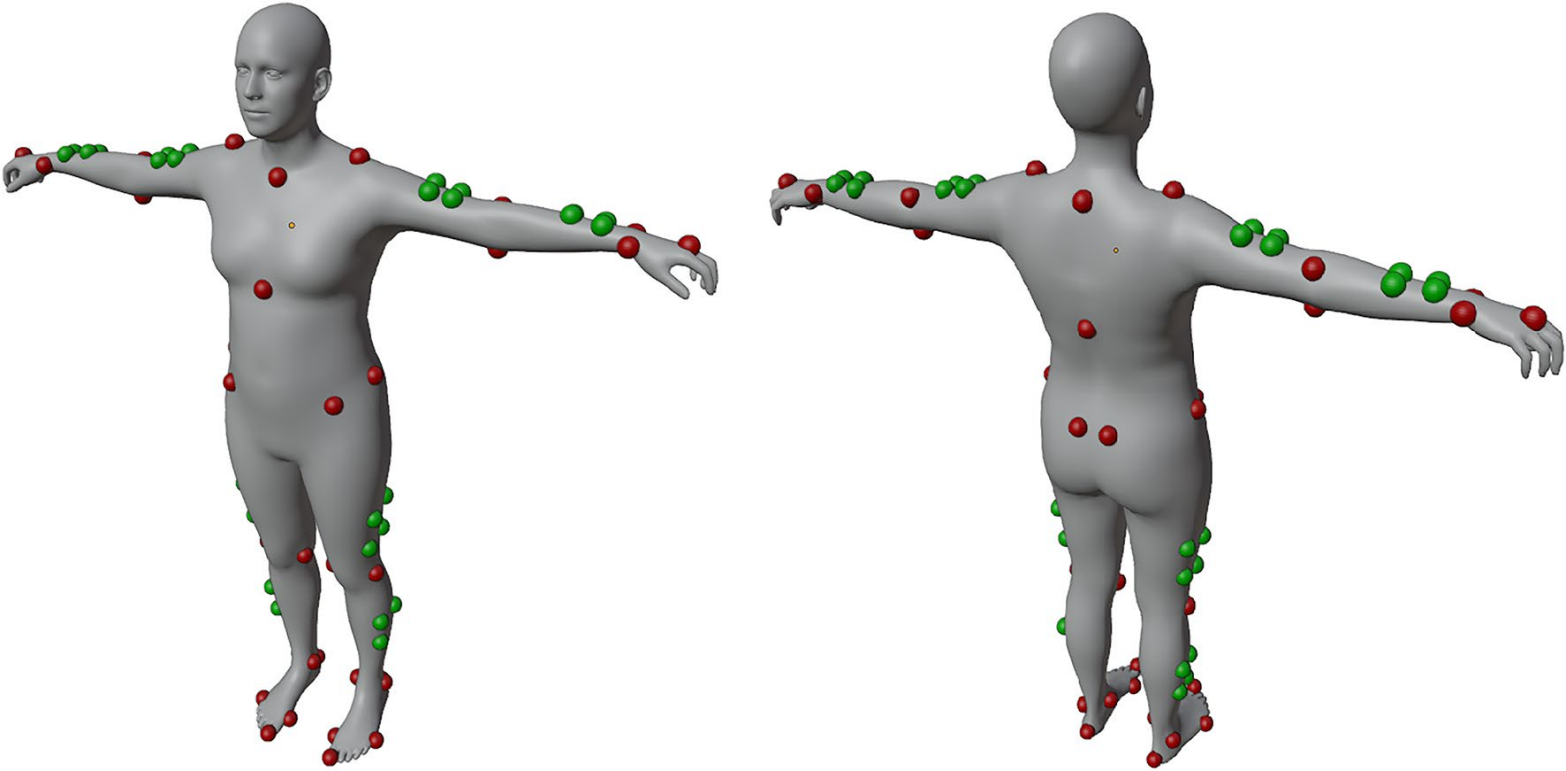
SMPL-X vertices were picked to match the original bioCV markers. Anatomical vertices are represented in red, cluster ones in green. Image generated with Blender 3.6 (https://www.blender.org).

We selected the vertices that were the closest to the bioCV marker positions (Fig. 6) and adapted SMPL2AddBiomechanics^29^ to output the position of the chosen SMPL-X vertices at each frame for each animated person. We also estimated heights and masses^30^: height was measured as the difference between the head top and heel points after setting the mesh in a T-pose, and mass was based on the volume of the mesh with an average density of 0.985.

In order to obtain a comparable sample to the bioCV dataset, we manually selected 16 CMJ, 12 RUN and 13 WALK trials performed by 22 different animated humans (65.1 ± 11.4 kg, 1.73 ± 0.07 m) at random from the dataset to test whether the findings from the bioCV dataset hold true for synthetic data. Foot strikes and the start and end of CMJ movement were manually detected.

### Model and marker registration/scaling

For the bioCV data, a static T-pose trial was used to scale an OpenSim^31,32^ rigid-lower-body model (consisting of 13 bones connected by 12 joints) to each participant’s anthropometrics. The hip and knee joint were modelled with three degrees of freedom, while the ankle and subtalar joint had a single degree of freedom^33,34^. The range of the knee joint and pelvis was constrained (Table 1). For scaling, joint centres were weighted at 100% and anatomical markers at 20% (the actual weight is irrelevant, since only the relative proportion is taken into account). We achieved average scaling errors of 7.8 ± 2.1 mm (total squared error), 17.8 ± 2.7 mm (root-mean squared error), and 31.0 ± 4.3 mm (maximum marker error). All scaled models were manually scaled, marker positions visually compared with the original marker positions, and joint angles checked for plausibility.

For the BEDLAM data, no static trials were available. Therefore, the two frames with least movement determined as the smallest absolute value of the sum of the derivative of all available markers per frame of each trial were selected to scale the same OpenSim rigid body model with the same scaling parameters as for the bioCV data. The synthetic markers of these frames were translated to the origin of the coordinate system and rotated to be in accordance with the OpenSim coordinate system. We achieved average scaling errors of 6.8 ± 1.7 mm (total squared error), 14.4 ± 1.3 mm (root-mean squared error), and 21.9 ± 6.4 mm (maximum marker error).

For the following IK calculations, the same scaled models were used for each marker combination. We thereby exclude error between marker combinations due to differences in scaling^9,10^.

### Inverse kinematics

**Fig. 7:**
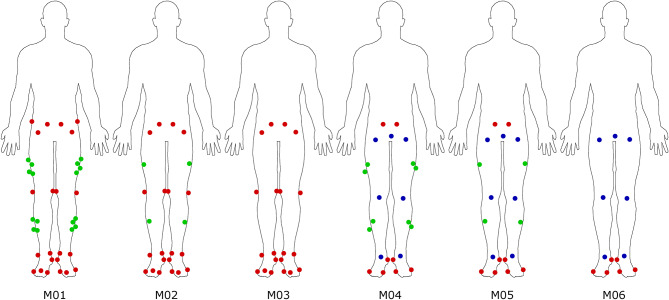
Overview of the different marker combinations tested. Anatomical markers are displayed in red, mid-segment markers in green and joint centres and mid-points in blue.

All dynamic trials were pre-processed in Python (version 3.11) prior to running OpenSim’s inverse kinematics tool (version 4.5). Inverse kinematics were calculated using multiple combinations of equally weighted markers to analyse their influence on the results (Table 1). We selected a gold-standard lower-body marker set (M01) containing anatomical and mid-segment markers for each segment as reference and systematically reduced these to match the number and location of commonly available pose estimation keypoints. M02 included anatomical and a single mid-segment marker, which allowed us to analyse the influence of redundant markers on the IK results. M03 included only anatomical markers to analyse the impact of mid-segment markers. M04 and M05 used the common pose estimation joint centre keypoints plus additional mid-segment markers; two for M04 and one for M05 respectively. Thereby, the number of markers was similar to M02 and M03, but in different locations. M06 contained the commonly available pose estimation keypoints only (Fig. 7). With this approach, we could define the minimum number and best location of markers needed for accurate IK results.

Joint angles were calculated using an XYZ Cardan sequence, with the hip, knee and ankle joint considered for analysis. The angle time-series were filtered using a second-order low-pass Butterworth filter with a cut-off frequency of 6 Hz. For right foot strikes, only the angles of the right side were analysed and for left foot strikes angles of the left side accordingly. For CMJ, angles of the left and right side were combined. A total of 994 time-normalised motion time-series from the bioCV dataset and 132 for the BEDLAM dataset were included.

### Analysis

The influence of the marker set was assessed using the RMSE for each time step and the mean RMSE between each marker combination (M02-M06) and the “ground truth” (M01). We further calculated the inter-protocol coefficient of multiple correlation (CMC)^35^ and the range of motion (ROM) for each joint angle. The CMC was interpreted as *<* 0.4 poor; 0.4–0.6 fair; 0.6–0.8 moderate; 0.8-1 good agreement. Linear mixed-effect models were fitted in R (version 4.4.1) to examine the effects of marker combination and movement (fixed effects) on ROM and RMSE. Participant was included as a random effect to account for inter-individual variability. The effect size was evaluated and interpreted as *<* 0.2 trivial; 0.2–0.6 small; 0.6–1.2 moderate; 1.2-2.0 large; *>*2 very large^36^.

## Electronic supplementary material

Below is the link to the electronic supplementary material.

Supplementary Material 1

## Data Availability

The publicly-available datasets analysed during the current study are available in: Evans, M., Needham, L., Wade, L., Parsons, M., Colyer, S., McGuigan, P., Bilzon, J., Cosker, D., 2024. BioCV Motion Capture Dataset. Bath: University of Bath Research Data Archive. Available from: 10.15125/BATH-01258. M.J. Black, P. Patel, J. Tesch, and J. Yang, BEDLAM: A Synthetic Dataset of Bodies Exhibiting Detailed Lifelike Animated Motion, in Proceedings IEEE/CVF Conf. on Computer Vision and Pattern Recognition (CVPR), Jun. 2023, pp. 8726–8737.
