# Supplementary material for: The influence of the marker set on inverse kinematics results to inform markerless motion capture annotations
Source: Sci Rep. 2025 Apr 25;15:14547. doi: 10.1038/s41598-025-97219-5 (PMC12032346; doi:10.1038/s41598-025-97219-5)
Supplement: Supplementary file 1 — Supplementary Material 1 [file 41598_2025_97219_MOESM1_ESM.pdf]

Supplementary information

Table S1. Mean and standard deviation of the RMSE and CMC values for all marker sets per movement and angle in the bioCV dataset (RMSE: green <2, yellow 2-5, red >5; CMC green good, orange moderate, yellow fair, red poor).

			RMSE mean					RMSE std					CMC				
BIOCV			M02	M03	M04	M05	M06	M02	M03	M04	M05	M06	M02	M03	M04	M05	M06
hip	flexion/ extension	CMJ	0.98	1.24	3.73	3.64	22.12	0.54	0.30	2.10	2.28	6.26	1.000	0.999	0.992	0.992	0.878
		RUN	0.91	1.18	2.04	1.88	23.02	0.39	0.42	1.13	1.11	8.35	0.999	0.999	0.995	0.996	0.683
		WALK	1.11	1.43	1.93	1.91	17.26	0.39	0.46	0.98	0.93	8.92	0.999	0.998	0.996	0.996	0.723
	adduction/ abduction	CMJ	0.37	0.39	0.50	0.59	0.90	0.17	0.17	0.20	0.21	0.34	0.996	0.995	0.993	0.990	0.976
		RUN	0.81	0.84	1.51	1.47	1.98	0.32	0.27	0.51	0.53	1.13	0.993	0.991	0.976	0.973	0.939
		WALK	0.73	0.74	1.23	1.13	2.20	0.22	0.26	0.40	0.38	2.76	0.995	0.994	0.987	0.988	0.905
	internal/ external rotation	CMJ	2.30	5.80	2.68	3.99	11.10	1.47	2.45	1.32	1.67	3.36	0.907	0.773	0.866	0.785	0.220
		RUN	2.22	4.78	3.92	4.49	11.66	0.86	2.39	1.35	1.52	5.82	0.863	0.518	0.699	0.716	
		WALK	1.85	3.64	3.17	4.04	16.07	0.85	2.45	1.25	1.55	9.25	0.924	0.676	0.827	0.802	
knee	flexion/ extension	CMJ	1.25	2.22	1.03	1.46	3.35	0.93	1.33	0.66	0.91	1.33	0.999	0.998	1.000	0.999	0.995
		RUN	0.96	1.55	1.08	1.30	2.71	0.53	0.87	0.62	0.83	1.26	0.999	0.999	0.999	0.999	0.996
		WALK	0.70	1.05	0.90	1.18	2.27	0.51	0.76	0.57	0.72	1.65	1.000	0.999	0.999	0.999	0.995
	adduction/ abduction	CMJ	2.71	6.35	2.01	3.24	8.22	2.07	2.80	0.94	1.10	2.26	0.532		0.904	0.851	0.375
		RUN	1.98	5.18	1.97	2.69	9.52	1.29	2.57	1.36	1.37	2.74	0.760		0.768	0.771	
		WALK	1.08	2.70	1.80	2.14	8.25	0.74	1.96	0.97	1.29	2.30	0.900	0.440	0.773	0.724	
	internal/ external rotation	CMJ	2.52	5.90	1.86	2.68	11.35	0.73	1.80	0.59	1.18	4.22	0.962	0.679	0.985	0.969	0.639
		RUN	3.01	6.29	1.89	3.18	10.00	1.31	2.86	0.51	0.80	3.22	0.875	0.358	0.968	0.924	0.481
		WALK	2.10	4.84	1.35	2.47	12.59	1.01	2.60	0.62	1.01	4.22	0.871	0.506	0.957	0.846	
ankle	plantar/ dorsi flexion	CMJ	0.59	0.84	1.56	1.66	1.80	0.53	0.62	0.71	0.75	0.92	1.000	0.999	0.998	0.998	0.997
		RUN	0.76	1.16	2.68	2.71	2.73	0.32	0.54	1.16	1.18	1.58	0.999	0.997	0.986	0.986	0.983
		WALK	0.57	0.91	2.95	3.00	3.07	0.45	0.62	1.05	1.12	1.34	0.997	0.994	0.959	0.957	0.955
	inversion/ eversion	CMJ	2.68	4.73	2.27	2.12	6.56	1.01	1.67	0.66	0.83	3.38	0.920	0.720	0.947	0.944	0.667
		RUN	2.96	6.31	2.60	2.79	5.19	1.51	3.28	1.27	0.73	1.98	0.843	0.183	0.916	0.924	0.783
		WALK	2.14	4.52	2.71	2.68	4.55	1.02	2.06	1.56	1.32	1.32	0.889	0.482	0.854	0.863	0.682
		MEAN	1.55	3.11	2.06	2.44	8.27	0.80	1.48	0.94	1.05	3.33	0.926	0.786	0.931	0.916	0.783

Table S2. Effect sizes for the RMSE of all marker sets per movement and angle in the bioCV dataset (brightness of blue trivial, small, moderate, large, very large).

BIOCV RMSE			M02-M03	M02-M04	M02-M05	M02-M06	M03-M04	M03-M05	M03-M06	M04-M05	M04-M06	M05-M06
hip	flexion/ extension	CMJ	0.1	0.8	0.8	6.1	0.7	0.7	6.1	0.0	5.3	5.4
		COMBINED	0.1	0.5	0.4	5.7	0.4	0.3	5.7	0.0	5.3	5.3
		RUN	0.1	0.3	0.3	6.4	0.3	0.2	6.3	0.0	6.1	6.1
		WALK	0.1	0.2	0.2	4.7	0.1	0.1	4.6	0.0	4.4	4.4
	adduction/ abduction	CMJ	0.0	0.1	0.2	0.6	0.1	0.2	0.5	0.1	0.4	0.3
		COMBINED	0.0	0.5	0.5	1.1	0.4	0.4	1.1	0.0	0.6	0.7
		RUN	0.0	0.7	0.7	1.2	0.7	0.7	1.2	0.0	0.5	0.5
		WALK	0.0	0.5	0.4	1.5	0.5	0.4	1.5	0.1	1.0	1.1
	internal/ external rotation	CMJ	1.0	0.1	0.5	2.4	0.9	0.5	1.5	0.4	2.3	2.0
		COMBINED	0.7	0.3	0.6	3.0	0.4	0.2	2.3	0.3	2.7	2.4
		RUN	0.7	0.5	0.6	2.6	0.2	0.1	1.9	0.2	2.1	2.0
		WALK	0.5	0.4	0.6	3.9	0.1	0.1	3.4	0.2	3.6	3.3
knee	flexion/ extension	CMJ	1.2	0.3	0.3	2.6	1.5	0.9	1.4	0.5	2.9	2.4
		COMBINED	0.8	0.0	0.4	2.3	0.8	0.4	1.5	0.4	2.2	1.8
		RUN	0.7	0.2	0.4	2.2	0.6	0.3	1.5	0.3	2.0	1.8
		WALK	0.4	0.3	0.6	2.0	0.2	0.2	1.5	0.3	1.7	1.4
	adduction/ abduction	CMJ	2.3	0.4	0.3	3.5	2.8	2.0	1.2	0.8	4.0	3.2
		COMBINED	1.8	0.0	0.5	4.3	1.8	1.3	2.5	0.5	4.3	3.8
		RUN	2.0	0.0	0.5	4.8	2.1	1.6	2.8	0.5	4.8	4.4
		WALK	1.0	0.5	0.7	4.6	0.6	0.4	3.6	0.2	4.1	3.9
	internal/ external rotation	CMJ	1.7	0.3	0.1	4.3	2.0	1.6	2.7	0.4	4.7	4.3
		COMBINED	1.5	0.4	0.1	4.3	2.0	1.4	2.8	0.5	4.7	4.2
		RUN	1.6	0.6	0.1	3.4	2.2	1.5	1.8	0.6	4.0	3.4
		WALK	1.3	0.4	0.2	5.2	1.7	1.2	3.8	0.6	5.5	5.0
ankle	plantar/ dorsi flexion	CMJ	0.3	1.4	1.5	1.7	1.0	1.2	1.4	0.1	0.3	0.2
		COMBINED	0.5	2.5	2.6	2.7	2.0	2.1	2.2	0.1	0.2	0.1
		RUN	0.6	2.7	2.7	2.8	2.1	2.2	2.2	0.0	0.1	0.0
		WALK	0.5	3.3	3.4	3.5	2.9	2.9	3.0	0.1	0.2	0.1
	inversion/ eversion	CMJ	1.3	0.3	0.4	2.5	1.6	1.7	1.2	0.1	2.8	2.9
		COMBINED	1.7	0.0	0.0	1.9	1.7	1.7	0.2	0.0	1.9	1.9
		RUN	2.2	0.2	0.1	1.5	2.4	2.3	0.7	0.1	1.7	1.6
		WALK	1.6	0.4	0.4	1.6	1.2	1.2	0.0	0.0	1.2	1.2

Table S3. Effect sizes for the ROM of all marker sets per movement and angle in the bioCV dataset (brightness of blue trivial, small, moderate, large, very large).

BioCV ROM			M01-M02	M01-M03	M01-M04	M01-M05	M01-M06	M02-M03	M02-M04	M02-M05	M02-M06	M03-M04	M03-M05	M03-M06	M04-M05	M04-M06	M05-M06
hip	flexion/ extension	CMJ	0.1	0.1	1.1	1.0	1.8	0.2	1.0	0.9	1.9	1.1	1.1	1.7	0.1	2.8	2.8
		COMBINED	0.1	0.0	0.4	0.4	1.5	0.1	0.4	0.4	1.6	0.4	0.4	1.5	0.0	2.0	2.0
		RUN	0.0	0.0	0.2	0.2	2.2	0.0	0.2	0.2	2.2	0.2	0.2	2.2	0.0	2.4	2.4
		WALK	0.1	0.1	0.0	0.1	0.7	0.0	0.0	0.0	0.7	0.0	0.0	0.7	0.0	0.7	0.7
	adduction/ abduction	CMJ	0.0	0.0	0.1	0.0	0.0	0.0	0.1	0.0	0.0	0.1	0.0	0.0	0.1	0.1	0.0
		COMBINED	0.1	0.1	0.2	0.1	0.1	0.3	0.1	0.2	0.3	0.3	0.1	0.0	0.3	0.3	0.1
		RUN	0.2	0.2	0.3	0.1	0.4	0.4	0.1	0.3	0.6	0.5	0.1	0.2	0.4	0.7	0.3
		WALK	0.2	0.2	0.3	0.1	0.0	0.4	0.0	0.3	0.2	0.4	0.1	0.2	0.3	0.2	0.1
	internal/ external rotation	CMJ	0.4	2.2	0.0	0.2	2.4	1.7	0.5	0.2	1.9	2.2	1.9	0.2	0.3	2.4	2.1
		COMBINED	0.3	1.0	0.4	0.9	2.1	0.7	0.1	0.6	1.8	0.6	0.1	1.1	0.5	1.7	1.2
		RUN	0.0	0.4	0.5	1.1	1.5	0.4	0.5	1.1	1.5	0.1	0.7	1.0	0.6	0.9	0.4
		WALK	0.4	0.5	0.7	1.3	2.4	0.0	0.3	0.9	2.0	0.2	0.8	2.0	0.6	1.8	1.2
knee	flexion/ extension	CMJ	0.2	0.6	0.1	0.2	0.8	0.4	0.3	0.4	0.6	0.7	0.8	0.2	0.1	0.9	1.0
		COMBINED	0.1	0.4	0.1	0.1	0.5	0.2	0.2	0.2	0.4	0.5	0.5	0.1	0.0	0.6	0.6
		RUN	0.1	0.2	0.1	0.1	0.3	0.2	0.2	0.2	0.2	0.3	0.3	0.1	0.0	0.4	0.4
		WALK	0.2	0.3	0.0	0.1	0.5	0.2	0.2	0.1	0.3	0.3	0.2	0.1	0.1	0.5	0.4
	adduction/ abduction	CMJ	0.9	1.8	1.4	2.7	4.6	0.8	0.4	1.7	3.6	0.4	0.9	2.8	1.3	3.2	1.9
		COMBINED	0.4	1.1	0.6	1.4	2.5	0.7	0.1	0.9	2.1	0.6	0.2	1.4	0.8	1.9	1.1
		RUN	0.5	1.3	0.4	1.2	1.7	0.9	0.0	0.7	1.3	0.9	0.1	0.4	0.8	1.3	0.5
		WALK	0.1	0.3	0.1	0.3	1.2	0.4	0.0	0.4	1.3	0.4	0.0	1.0	0.4	1.3	1.0
	internal/ external rotation	CMJ	0.2	0.5	0.9	1.2	3.0	0.6	0.7	1.0	2.9	1.4	1.7	3.5	0.3	2.1	1.8
		COMBINED	0.0	0.2	0.6	0.8	2.3	0.1	0.6	0.8	2.3	0.7	1.0	2.4	0.2	1.7	1.5
		RUN	0.1	0.1	0.8	1.3	2.4	0.2	0.7	1.3	2.4	0.9	1.5	2.6	0.5	1.6	1.1
		WALK	0.3	0.1	0.0	0.1	1.4	0.5	0.3	0.2	1.7	0.2	0.3	1.2	0.1	1.4	1.5
ankle	plantar/ dorsi flexion	CMJ	0.1	0.1	0.0	0.1	0.2	0.0	0.1	0.2	0.1	0.1	0.2	0.0	0.1	0.2	0.3
		COMBINED	0.2	0.1	0.1	0.1	0.0	0.0	0.2	0.3	0.1	0.2	0.3	0.1	0.0	0.1	0.2
		RUN	0.2	0.2	0.0	0.1	0.4	0.1	0.2	0.2	0.1	0.1	0.1	0.2	0.0	0.3	0.3
		WALK	0.1	0.1	0.3	0.3	0.5	0.0	0.4	0.4	0.6	0.4	0.4	0.5	0.0	0.1	0.1
	inversion/ eversion	CMJ	0.2	0.4	0.4	0.1	2.0	0.6	0.2	0.1	1.9	0.8	0.5	2.5	0.3	1.6	1.9
		COMBINED	0.1	0.5	0.4	0.4	1.4	0.4	0.5	0.5	1.4	0.9	0.9	1.8	0.0	0.9	0.9
		RUN	0.0	0.3	0.8	1.1	1.9	0.3	0.8	1.1	1.9	1.1	1.4	2.2	0.3	1.1	0.8
		WALK	0.4	0.7	0.1	0.1	0.2	0.3	0.5	0.5	0.6	0.8	0.8	0.8	0.0	0.1	0.1

Table S4. Mean and standard deviation of the RMSE and CMC values for all marker sets per movement and angle in the synthetic BEDLAM dataset (RMSE: green <2, yellow 2-5, red >5; CMC green good, orange moderate, yellow fair, red poor).

			RMSE mean					RMSE std					CMC				
BEDLAM			M02	M03	M04	M05	M06	M02	M03	M04	M05	M06	M02	M03	M04	M05	M06
hip	flexion/ extension	CMJ	1.42	1.26	2.55	2.60	17.19	0.75	0.72	1.34	1.47	4.60	0.999	0.999	0.995	0.994	0.850
		RUN	0.83	0.99	1.60	1.61	20.68	0.59	0.68	0.80	0.84	9.39	0.998	0.997	0.994	0.993	0.564
		WALK	1.22	1.38	1.72	1.74	22.27	0.44	0.54	0.78	0.79	11.81	0.998	0.998	0.996	0.996	0.716
	adduction/ abduction	CMJ	0.51	0.57	0.74	0.78	1.29	0.20	0.18	0.48	0.47	0.73	0.992	0.991	0.981	0.980	0.944
		RUN	0.81	0.82	1.11	1.50	2.82	0.33	0.21	0.33	0.57	1.45	0.985	0.984	0.972	0.943	0.805
		WALK	0.89	0.93	1.32	1.32	3.03	0.28	0.21	0.62	0.69	2.17	0.989	0.988	0.973	0.970	0.776
	internal/ external rotation	CMJ	1.86	3.60	3.03	3.59	9.18	0.72	2.02	1.57	2.08	4.88	0.957	0.883	0.752	0.728	0.171
		RUN	1.57	2.91	4.43	5.86	18.14	1.12	1.66	1.75	2.99	7.30	0.938	0.859	0.544	0.186	0.489
		WALK	1.83	3.50	4.05	4.73	12.05	0.78	2.90	4.10	3.66	5.66	0.931	0.762	0.325	0.365	0.196
knee	flexion/ extension	CMJ	0.99	1.40	1.40	1.30	2.55	0.39	0.43	0.52	0.57	1.13	1.000	0.999	0.999	0.999	0.996
		RUN	0.75	0.90	1.30	1.39	2.99	0.33	0.36	0.74	0.85	1.93	1.000	0.999	0.999	0.998	0.992
		WALK	0.72	1.03	1.32	1.33	2.09	0.20	0.44	1.11	1.23	1.51	1.000	0.999	0.998	0.998	0.995
	adduction/ abduction	CMJ	2.15	4.14	2.34	3.19	6.66	1.07	2.87	1.57	2.03	3.64	0.901	0.650	0.622	0.626	0.133
		RUN	1.46	3.07	1.89	2.94	6.09	1.11	1.63	1.63	2.74	2.86	0.875	0.618	0.838	0.611	
		WALK	1.28	2.84	1.54	2.17	5.32	0.68	2.89	1.06	1.02	3.18	0.951	0.614	0.908	0.878	0.253
	internal/ external rotation	CMJ	2.12	3.79	1.79	2.85	10.14	0.67	1.35	0.98	1.36	3.61	0.958	0.878	0.971	0.943	0.769
		RUN	1.93	3.57	1.26	2.52	8.96	0.88	1.44	0.49	1.79	3.79	0.917	0.765	0.970	0.861	0.291
		WALK	2.08	3.89	1.67	3.24	10.19	0.76	1.21	1.40	1.58	3.60	0.949	0.826	0.957	0.883	
ankle	plantar/ dorsi flexion	CMJ	0.57	0.85	1.24	1.43	2.37	0.26	0.41	0.40	0.37	1.09	1.000	0.999	0.999	0.999	0.995
		RUN	0.70	1.03	1.38	1.69	2.57	0.33	0.38	0.40	0.51	0.85	0.997	0.994	0.989	0.984	0.959
		WALK	0.43	0.81	1.41	1.50	1.81	0.21	0.35	1.07	1.04	0.89	0.998	0.994	0.978	0.976	0.968
	inversion/ eversion	CMJ	2.26	4.23	2.55	3.66	7.48	0.96	1.84	1.61	1.87	3.93	0.931	0.772	0.868	0.772	0.642
		RUN	2.64	4.38	3.34	3.96	5.63	1.23	1.89	1.51	1.47	2.63	0.878	0.658	0.830	0.754	0.472
		WALK	1.91	4.29	2.72	2.70	5.59	0.74	1.49	1.78	1.23	1.47	0.932	0.532	0.866	0.880	0.484
		MEAN	1.37	2.34	1.99	2.48	7.79	0.63	1.17	1.17	1.38	3.50	0.961	0.865	0.889	0.847	0.657

Table S5. Effect sizes for the RMSE of all marker sets per movement and angle in the synthetic BEDLAM dataset (brightness of blue trivial, small, moderate, large, very large).

BEDLAM RMSE			M02-M03	M02-M04	M02-M05	M02-M06	M03-M04	M03-M05	M03-M06	M04-M05	M04-M06	M05-M06
hip	flexion/ extension	CMJ	0.0	0.3	0.3	3.9	0.3	0.3	3.9	0.0	3.6	3.6
		COMBINED	0.0	0.2	0.2	4.6	0.2	0.2	4.6	0.0	4.4	4.4
		RUN	0.0	0.2	0.2	4.9	0.1	0.2	4.8	0.0	4.7	4.7
		WALK	0.0	0.1	0.1	5.2	0.1	0.1	5.1	0.0	5.0	5.0
	adduction/ abduction	CMJ	0.1	0.3	0.4	1.1	0.2	0.3	1.0	0.1	0.8	0.7
		COMBINED	0.1	0.4	0.6	2.2	0.4	0.6	2.2	0.2	1.8	1.6
		RUN	0.0	0.4	1.0	2.8	0.4	0.9	2.7	0.5	2.3	1.8
		WALK	0.1	0.6	0.6	2.9	0.5	0.5	2.9	0.0	2.3	2.3
	internal/ external rotation	CMJ	0.5	0.4	0.5	2.2	0.2	0.0	1.7	0.2	1.9	1.7
		COMBINED	0.5	0.6	0.9	3.5	0.2	0.4	3.0	0.3	2.8	2.6
		RUN	0.4	0.9	1.3	5.1	0.5	0.9	4.7	0.4	4.2	3.8
		WALK	0.5	0.7	0.9	3.1	0.2	0.4	2.6	0.2	2.4	2.2
knee	flexion/ extension	CMJ	0.5	0.5	0.3	1.8	0.0	0.1	1.3	0.1	1.3	1.4
		COMBINED	0.3	0.6	0.6	1.9	0.3	0.3	1.6	0.0	1.4	1.4
		RUN	0.2	0.6	0.7	2.5	0.4	0.5	2.4	0.1	1.9	1.8
		WALK	0.3	0.7	0.7	1.5	0.3	0.3	1.2	0.0	0.9	0.8
	adduction/ abduction	CMJ	1.0	0.1	0.5	2.3	0.9	0.5	1.3	0.4	2.2	1.8
		COMBINED	0.9	0.2	0.6	2.3	0.7	0.3	1.4	0.4	2.1	1.7
		RUN	0.8	0.2	0.8	2.4	0.6	0.1	1.6	0.5	2.2	1.6
		WALK	0.8	0.1	0.5	2.1	0.7	0.3	1.3	0.3	1.9	1.6
	internal/ external rotation	CMJ	0.9	0.2	0.4	4.1	1.0	0.5	3.2	0.5	4.3	3.7
		COMBINED	0.9	0.2	0.4	3.9	1.1	0.4	3.1	0.7	4.2	3.5
		RUN	0.8	0.3	0.3	3.6	1.2	0.5	2.8	0.6	3.9	3.3
		WALK	0.9	0.2	0.6	4.1	1.1	0.3	3.2	0.8	4.4	3.6
ankle	plantar/ dorsi flexion	CMJ	0.5	1.2	1.6	3.3	0.7	1.1	2.8	0.4	2.1	1.7
		COMBINED	0.6	1.4	1.8	3.1	0.8	1.2	2.5	0.4	1.7	1.3
		RUN	0.6	1.3	1.8	3.4	0.6	1.2	2.8	0.6	2.2	1.6
		WALK	0.7	1.8	2.0	2.5	1.1	1.3	1.8	0.2	0.7	0.6
	inversion/ eversion	CMJ	1.2	0.2	0.8	3.1	1.0	0.3	1.9	0.6	2.9	2.2
		COMBINED	1.2	0.4	0.7	2.3	0.8	0.5	1.1	0.3	2.0	1.6
		RUN	1.0	0.4	0.8	1.8	0.6	0.3	0.7	0.4	1.3	1.0
		WALK	1.4	0.5	0.5	2.2	0.9	0.9	0.8	0.0	1.7	1.7

Table S6. Effect sizes for the ROM of all marker sets per movement and angle in the synthetic BEDLAM dataset (brightness of blue trivial, small, moderate, large, very large).

BEDLAM ROM			M01-M02	M01-M03	M01-M04	M01-M05	M01-M06	M02-M03	M02-M04	M02-M05	M02-M06	M03-M04	M03-M05	M03-M06	M04-M05	M04-M06	M05-M06
hip	flexion/ extension	CMJ	0.2	0.1	0.5	0.5	0.2	0.1	0.3	0.3	0.4	0.4	0.4	0.3	0.0	0.7	0.7
		COMBINED	0.1	0.1	0.2	0.2	1.0	0.0	0.1	0.1	1.1	0.1	0.1	1.1	0.0	1.2	1.2
		RUN	0.0	0.0	0.1	0.1	0.9	0.0	0.0	0.0	0.9	0.1	0.1	0.9	0.0	1.0	0.9
		WALK	0.1	0.1	0.0	0.0	1.9	0.0	0.0	0.0	2.0	0.0	0.0	2.0	0.0	2.0	2.0
	adduction/ abduction	CMJ	0.0	0.0	0.0	0.1	0.2	0.0	0.0	0.0	0.1	0.0	0.1	0.2	0.0	0.1	0.1
		COMBINED	0.1	0.0	0.0	0.1	0.0	0.1	0.1	0.2	0.1	0.0	0.1	0.1	0.1	0.0	0.1
		RUN	0.2	0.0	0.0	0.2	0.4	0.2	0.2	0.3	0.3	0.0	0.2	0.4	0.1	0.4	0.6
		WALK	0.2	0.1	0.1	0.1	0.1	0.2	0.1	0.2	0.3	0.2	0.0	0.1	0.2	0.2	0.1
	internal/ external rotation	CMJ	0.3	0.9	0.3	0.1	0.7	0.5	0.6	0.4	0.3	1.1	1.0	0.2	0.2	1.0	0.8
		COMBINED	0.3	0.7	0.1	0.1	2.1	0.4	0.4	0.2	1.8	0.7	0.6	1.4	0.1	2.1	2.0
		RUN	0.2	0.5	0.1	0.2	3.8	0.2	0.2	0.1	3.5	0.4	0.3	3.3	0.1	3.7	3.6
		WALK	0.3	0.6	0.0	0.2	1.8	0.3	0.3	0.1	1.5	0.6	0.5	1.1	0.1	1.7	1.6
knee	flexion/ extension	CMJ	0.2	0.2	0.0	0.1	0.7	0.0	0.1	0.1	0.5	0.2	0.1	0.5	0.0	0.7	0.6
		COMBINED	0.1	0.2	0.0	0.1	0.3	0.0	0.1	0.1	0.2	0.2	0.1	0.2	0.0	0.3	0.3
		RUN	0.1	0.1	0.0	0.1	0.1	0.0	0.0	0.0	0.0	0.1	0.0	0.0	0.0	0.1	0.0
		WALK	0.1	0.2	0.1	0.0	0.2	0.1	0.2	0.1	0.1	0.3	0.2	0.0	0.1	0.3	0.2
	adduction/ abduction	CMJ	0.9	1.1	0.4	0.6	1.1	0.1	1.3	0.3	0.2	1.5	0.5	0.0	1.0	1.5	0.5
		COMBINED	0.5	0.6	0.0	0.5	0.8	0.1	0.5	0.0	0.3	0.6	0.1	0.2	0.5	0.8	0.3
		RUN	0.0	0.1	0.3	0.3	0.4	0.1	0.3	0.3	0.5	0.2	0.2	0.3	0.0	0.2	0.1
		WALK	0.5	0.5	0.1	0.5	0.8	0.1	0.4	0.0	0.4	0.5	0.0	0.3	0.4	0.7	0.3
	internal/ external rotation	CMJ	0.2	0.6	0.5	0.9	3.7	0.3	0.3	0.6	3.5	0.1	0.3	3.2	0.4	3.2	2.8
		COMBINED	0.0	0.2	0.3	0.4	1.9	0.1	0.2	0.4	1.9	0.1	0.3	1.7	0.2	1.6	1.5
		RUN	0.0	0.1	0.2	0.4	1.7	0.1	0.2	0.3	1.7	0.1	0.2	1.6	0.2	1.5	1.4
		WALK	0.2	0.2	0.1	0.0	0.2	0.0	0.3	0.2	0.4	0.2	0.2	0.4	0.0	0.1	0.2
ankle	plantar/ dorsi flexion	CMJ	0.0	0.0	0.0	0.1	0.2	0.0	0.0	0.0	0.1	0.0	0.1	0.2	0.0	0.1	0.1
		COMBINED	0.1	0.0	0.0	0.1	0.0	0.1	0.1	0.2	0.1	0.0	0.1	0.1	0.1	0.0	0.1
		RUN	0.2	0.0	0.0	0.2	0.4	0.2	0.2	0.3	0.3	0.0	0.2	0.4	0.1	0.4	0.6
		WALK	0.2	0.1	0.1	0.1	0.1	0.2	0.1	0.2	0.3	0.2	0.0	0.1	0.2	0.2	0.1
	inversion/ eversion	CMJ	0.1	0.3	0.3	0.3	2.0	0.2	0.4	0.4	1.9	0.6	0.6	1.7	0.0	2.3	2.3
		COMBINED	0.2	0.3	0.1	0.2	0.6	0.1	0.0	0.0	0.7	0.2	0.1	0.9	0.1	0.7	0.7
		RUN	0.3	0.5	0.1	0.3	0.1	0.1	0.2	0.1	0.2	0.4	0.2	0.3	0.1	0.0	0.1
		WALK	0.2	0.7	0.1	0.0	0.2	0.5	0.3	0.2	0.1	0.8	0.7	0.6	0.1	0.2	0.1
